# Policing Farm Animal Welfare in Federated Nations: The Problem of Dual Federalism in Canada and the USA

**DOI:** 10.3390/ani3041086

**Published:** 2013-12-02

**Authors:** Terry L. Whiting

**Affiliations:** Office of the Chief Veterinarian, Manitoba Agriculture Food and Rural Initiatives, 545 University Crescent, Winnipeg, Manitoba, Canada R3T 5S6; E-Mail: terry.whiting@gov.mb.ca; Tel.: +1-204-945-6750; Fax: +1-204-945-4327

**Keywords:** animal law, Criminal law, law enforcement, policing, Christian inquisition, SPCA, method of production, animal cruelty, division of powers, fundamentalism, zealot

## Abstract

**Simple Summary:**

In any federation of states, societal oversight of farm animal welfare (agriculture policy arena, prevention) is more difficult to achieve than providing punishment of individuals abusing of companion animals (post injury). The constitutional division of powers and historical policy related to animal agriculture and non-government organization policing cruelty of companion animals may be entrenched. With changing societal expectations of agriculture production, each level of government may hesitate to take the lead, due to financial or ideological beliefs and simultaneously, obstruct the other government level from taking the lead, based on constitutional grounds. The tradition of private policing of companion animal abuse offences may be unworkable in the provision of protection for animals used in industrial production.

**Abstract:**

In recent European animal welfare statutes, human actions injurious to animals are new “offences” articulated as an injury to societal norms in addition to property damage. A crime is foremost a violation of a community moral standard. Violating a societal norm puts society out of balance and justice is served when that balance is returned. Criminal law normally requires the presence of *mens rea*, or evil intent, a particular state of mind*;* however, dereliction of duties towards animals (or children) is usually described as being of varying levels of negligence but, rarely can be so egregious that it constitutes criminal societal injury. In instrumental justice, the “public goods” delivered by criminal law are commonly classified as retribution, incapacitation and general deterrence. Prevention is a small, if present, outcome of criminal justice. Quazi-criminal law intends to establish certain expected (moral) standards of human behavior where by statute, the obligations of one party to another are clearly articulated as strict liability. Although largely moral in nature, this class of laws focuses on achieving compliance, thereby resulting in prevention. For example, protecting the environment from degradation is a benefit to society; punishing non-compliance, as is the application of criminal law, will not prevent the injury. This paper will provide evidence that the integrated meat complex of Canada and the USA is not in a good position to make changes to implement a credible farm animal protection system.

## 1. Introduction

The primary focus of this paper is to review the problem of providing regulatory oversight and assurance of the welfare of farm and commercial animals in the Canadian and US context. The paper is organized around five main domains; (1) What constitutes reasonable, acceptable and possible societal oversight of the welfare of animals used in food and fiber production; (2) What aspects of Political Anthropology specific to North America is pertinent to achieving or preventing “reasonable and acceptable societal oversight”; (3) The problem of division of powers inherent in dual-federalism as they have evolved and manifest themselves in Canada and the USA; (4) The easily identified obstacles to achieving reasonable use of livestock animals; and (5) Possible directions for the future. 

Current animal protection legislative provisions are based on a blending of at least four separate moral principles: (a) animals should be protected from injury or death because of their status as property; (b) animals should be protected from unnecessary pain or injury in their own right as they have the capacity to suffer; (c) humans have a duty of care to animals; and (d) humans willfully causing animal injury is a morally repugnant human behavior [1]. 

The public concern with the treatment of animals primarily produced, used, and processed into animal products for human consumption as food and fiber is a social problem that cannot be solved by one level of government alone, by the industry alone, or by well meaning Non Profit Organizations (NPOs) alone. This paper is purely theoretical, as currently in North America farm animals have no legal protection from injury, pain or distress resulting from “common agricultural practices”. This paper will consider the problem of placing under regulatory oversight the production of animals for food or fiber. This is a paradigm shift from the current social norm where farmers are empowered to practice Cartesian principals of livestock production, to a new social norm where farmers are granted permission to farm when in compliance with the animal welfare standards acceptable to democratic society at large and the farm community supports new methods of production. This shift will be one of enormous scale considering the current state of industrialized livestock production in North America. 

## 2. Overview

Law, an articulation of the will of the populace, cannot be expressed without administrative machinery [2]. Legislative created bureaucracy generally establishes the relationship between a legally constituted and legitimate authority and its subordinate officials. In medieval England prior to emergence of “police”, maintaining the King’s Peace was a shire and a citizen responsibility. Industrialization, technological advancement and urbanization of modern societies have driven human specialization. Police; public, private and specialist have emerged as pivotal in enforcement of law in complex modern societies [3,4]. Traditional enforcement requires identifying an offence when and where it occurs; identifying the offender and bringing the matter before the judicial system in a way which follows the rules of natural justice. Natural justice is fair, transparent, and proportionate and respects fundamental human rights [5]. Evaluation of the consensus responsibility of humans to animals is complex and North American society has not yet approached a consensus when it comes to the fair and responsible use of farm animals. 

The specific social sanction of many brutal common farm animal practices such as hot iron branding, scoop dehorning, adult animal castration, trans-vaginal ovariectomy [6] all commonly practiced in Canada and the U.S. in the absence of pain mitigation, suggests farm animal vivisection remains the social norm. The standard common law test of criminal liability is usually expressed in the Latin phrase; *actus non facit reum nisi mens sit rea*, which means "the act is not culpable unless the mind is guilty". Articulating the sentience of animals, and the duty of care in animal welfare law, places certain human neglect as quazi-criminal where it violates the duty of care (*mens rea* not required) and some acts of human behavior are truly criminal, such as intentional injury of animals. For example, *The Animal Care Act* (Manitoba) [7,8] is quazi-criminal in that it places morally based obligations on individuals having control over animals and as an affirmative statute (*vs*. prohibitive statute) it applies the principal of strict liability. A strict liability offence is a type of offence that does not require any fault elements to be proved in order to establish guilt. The prosecution only needs to show that the accused failed to meet the statutory standard (e.g., cattle died of starvation while in the person’s possession). 

A recent study by the Food and Agricultural Organization (FAO), a division of The United Nations, provides an overview of how nations are dealing with the growing public pressure for increased oversight of the exploitation of animals for the benefit of a growing world human population [9]. The FAO document recognizes that the provision of animal protection requires a system involving (a) a competent authority; (b) a method of providing advice to the authority; (c) an operational arm of law enforcement; (d) laws describing standards to enforce; and (e) the ongoing engagement of civil society. The ongoing engagement of civil society is somewhat unique to animal welfare protection laws. The authors believe an established strong and dynamic institutional relationship between animal welfare scientists and regulatory agencies is an important precursor to good farm animal welfare legislation. The primary legislative objective is the establishment of a culture of respect for animal welfare and buttressing the symbiotic relation between animal welfare and human welfare [9]. This FAO statement also has significant aspects of policing-by-consent social theory. 

The Office des épizooties (OIE), an international Veterinary organization and standard setting contractor to the World Trade Organization (WTO), articulates the secular social norm “*the use of animals carries with it an ethical responsibility to ensure the welfare of such animals to the greatest extent practicable*” (Section 7 of the International Animal Health Code). In international trade in animals and animal products, future participation or leadership is to be expected from both the FAO and the OIE [10].

Animal protection law made major strides in the mid 1800s in Britain and North America. In early animal protection legislation the primary societal attitude driving change was concern regarding the moral state of the human actor rather than the suffering incurred by the animal. This was an expression of what came to be known as a new Victorian ethic. This focus on human behavior, and even more specifically on the malicious intent manifested as human behavior, placed early animal protection laws within criminal codes. Instrumentally the laws were focused on “punishing the offender” construct of justice. However, enforcement of emerging social standards in Victorian times was problematic as the setting of moral standards and informing the public of these standards was a new function of government. The enactment of the animal protection ethic preceded the development of uniformed police services. Previous to this era, policing largely focused on property protection and the personal citizen responsibility to “keep the King’s peace” had not generally been identified as a pseudo-communal (government) service contributing to the public good [11]. In the late 1800s there was limited intergovernmental relationships between Nation-State-County levels of governance and animal welfare law initially emerged at the level of city (municipal) [12].

Citizens have two methods of communicating convictions related to food animal use; either through market signals, not purchase the product; or, political action, pressure elected authorities to increase concern for the welfare of animals (e.g., Legislative Initiatives). Consumers can communicate directly with production chains by boycotting product or preferentially paying for specialty certified product. Consumers, who won’t voluntarily pay more for specific production practices as an individual, will often vote with non-consumers to make everyone pay more as demonstrated in recent political campaigns in the United States [13]. 

A minority of citizens, however, are not consumers (Vegan-vegetarian in this context) and cannot communicate their citizen conviction by purchase patterns. Citizen concerns are communicated by voting patterns and sometimes by participation or support of political agendas and non-profit political organizations. In addition Statist governments are often willing to constrain economic development in agriculture if supported by citizen concerns [14]. States with significant agriculture industry usually have an effective agriculture lobby and treat the farm industry in a corporatist (as a voting block); that is, the corporatist farmer vote is more based on identity of the farmer as a member of a group than the geographic elected representative who may represent some farmers in his/her riding. 

A study by Lusk and Norwood [15] in the USA suggested that people’s philosophic views on animal welfare are deeply embedded and not likely to be strongly influenced by education campaigns; especially if those current views are based in existing moral and ethical conviction. The attitudes of the population are not highly influenced by many science based measures of productivity and animal welfare. There is a theory that changing societal convictions on farming methods of production will foster market incentives for self-administered industry standards and minimize the need for societal based regulatory assurance [16]. In spite of some industry led humane slaughter assurance programs [17] there is however little physical evidence of significant consumer engagement in farm animal welfare in Canada and the USA.

Assuming that there will someday be a generally agreed to societal non-human animal exploitation threshold, below which are behaviors considered morally unacceptable for a human to participate in; the use of state coercion against the animal exploiter will be, morally justified, farmer supported and legally required. Enforcement, how state coercion is organized, delivered and controlled; who pays for the policing service and discourse on who should pay, provides some information on the culture of the resultant social organization. The approach of policing will also influence the probability of peaceful acceptance, compliance by consent, willingness to support the enforcement action and adoption by the individuals and corporations regulated.

## 3. Agro-Anthropology of the USA and Canada

Anthropology is the study of humankind, past and present, which draws and builds upon knowledge from social and biological sciences, as well as the humanities and the natural sciences. A central concern of anthropologists is the application of knowledge to the solution of human problems. In thinking of possible methods of encouraging a new social norm in the treatment and level of respect for farm production animals may be considered an attempt at social engineering [18]; thus, a review of the culture aspects of agriculture are in order.

### 3.1. Agriculture Exceptionalism

US Agricultural Policy is largely protectionist of domestic programs while aggressively seeking access to foreign markets. The USDA between its inception in 1862 and 1932 was a small agricultural science organization, maintaining agricultural statistics. Under Roosevelt, in 1932 it became the action arm of the “New Deal” and an instrument of national social planning. Also, in 1862, the Morrill Act provided a grant to each state of 30,000 acres of land for each representative and senator in congress to encourage the establishment and maintain an agricultural and mechanical college in each state, now referred to as “Land Grant Universities” [19]. Over the last 150 years the USDA and the Land Grant Universities have provided the government funding schemes and subsidies to agriculture that are allowable under World Trade Law. “Green Box” subsidy programs are not linked to volume of production so in theory does not distort trade [20]. Allowed programs include, subsidized research and extension, subsidized crop insurance, subsidized credit, subsidized conservation and environmental protection (grants), subsidized food programs (USDA’s Commodity Procurement Branch), marketing orders (e.g., peanut cartel), and tax preferences in agriculture [21,22]. In addition, U.S. farmers also have for many years held a “special” place of protection in American bankruptcy law [23].

The ideological neo-conservative right championed by the Thatcher-Regan policy era and currently ongoing, is based upon the strongly held faith that market individualism is the key to economic and social progress, and that the best outcomes for society will be realized when governments remove constraints from business activity and retreat from involvement in social and other programs that are viewed as both ‘distorting’ market signals and breeding dependency through subsidy payments [24]. This dogma, widely espoused by the Canadian and American elected representatives as official policy and universal truth (theology), made flesh by the North American Free Trade Agreement [25], has however, not received significant traction when applied to agriculture [20,26]. 

In North America as elsewhere, both national and state (sub-national) governments have an agriculture Ministry; few other sectors of the economy are given this prominence. The Doha round (2006–2007) of the World Trade Organization (WTO) ongoing global free trade initiative, collapsed largely because of the USA and the European Union (EU) reluctance to put farm tariffs and agriculture subsidies on the table for discussion, for example the $180 billion 2002 US Farm Bill, and the Common Agriculture Policy of the EU [27]. Agriculture policy has slowed liberalization of multilateral trade negotiations since the 1960s [22]. In a testament to the power of the U.S. farm lobby, fifty-seven out of one hundred U.S. Senators signed a letter pressing the then President George W. Bush not to offer any substantial reduction of farm subsidies in the Doha round of World Trade negotiations [21,28]. In pursuing the retention of the EU state assistance program (Common Agricultural Policy), the European farm lobby has been ascribed qualities of “asymmetrical interest, extraordinary organization, and remarkably biased enfranchisement” [29].

### 3.2. Agrarianism and Jefferersonian Democracy

Agrarianism as used in this discussion refers to a social or political philosophy (moral construct) which values rural society as superior to urban society; the independent farmer as superior to the paid worker; and sees farming as a way of life that can shape the ideal social values in the ideal citizen [30]. Thomas Jefferson built Jeffersonian Democracy around the notion that farmers are “the most valuable citizens” and the truest republicans.

*Cultivators of the earth are the most valuable citizens. They are the most vigorous, the most independent, the most virtuous, and they are tied to their country and wedded to its liberty and interests by the most lasting bonds*.

—*Thomas Jefferson 1785* (as quoted in Thompson 1990) [31]

Although often referred to as the “Agrarian Myth” this myth has made American agricultural policy very resilient to change and has extended into other areas of rural life such as retarding the implementation of basic human health and safety standards for agricultural workers [32]. In a survey of taxpayer preferences for farm policy, although there was overwhelming support for farm subsidies (85%) only 6.3% chose “to preserve a traditional lifestyle that has historical significance” [33]. This suggests that the agrarian ideal carries more weight in political rhetoric than it does in current social and political thought.

American Agrarianism, attachment to the land, and the magical properties of soil related hard work and ingenuity was fundamental to the vision of a individual driven democratic partnership which has maintained threads through current U.S. corporatist agricultural policy, which is heavily weighted to the crop and/or land based products over livestock and livestock products [30]. In the EU, the idea, or political construct of agriculture exceptionalism fosters and maintains the ‘state agriculture assistance paradigm’ of the Common Agricultural Policy (CAP) [21].

### 3.3. The US Farm Bill

Historically, the US economy is essentially pluralist and free-market in areas other than agriculture [34], supported by-neo-conservative rhetoric that unrestrained capitalism was a universal good. This is, with the exceptions of the recent bail out of the Big Automakers [35], and the bail out of several financial institutions subsequent to the global financial crisis related to subprime mortgage practices [36,37,38]. These undeniable pernicious manifestations of unrestrained capitalism have done little to dull the evangelical conviction of US and Canadian based schools of economics.

Farm policy has been largely driven by the 20th century convention that it was morally correct for society to protect small family farmers, because the vagaries of weather could cause devastation over which the farmer had no control; memories of the great depression and the distinctly American mythology created by the novels of John Steinbach. In a review of early post WWII farm policy debate (commodity prices always fall dramatically after major international conflict and trigger severe drop in value of farm products) Bradley effectively describes how family farmers can be manipulated to accept the cognitive dissonance of a permanent corporatist subsidy by evoking the fantasy that farmers are in the “Farming Class” not the working class, and they are self-employed, self-reliant, entrepreneurs in a free-market never to be the recipient of “government welfare” when even a cursory examination of reality would suggest the opposite [39]. 

In agriculture, for cultural and political reasons, US farm subsidy programs must remain approaches of market manipulation to maintain intact the veneer of the yeoman image of the American Farmer. The U.S. taxpayer supports subsidies at levels which provide the farmer a higher than national average standard of living [33]. The current hero-farmer ideology would be contradicted by policies that suggest livestock producers require significant legislative oversight in methods of production. The basic US policy of agriculture subsidy via commodity price support (initially commodity purchase and storage), has been very resilient to change, even though it provides a disproportionate benefit the larger the farm operation, has promoted monoculture and industrialization of agriculture and is projected to exceed $90 billion (if you include school lunch programs, and other commodity buy up programs) in 2013. While only 0.34% of the U.S. population claims farming as their principal occupation [33], $17 billion direct farm support was paid in 2009 [20,40]. 

European citizen organizations politically opposed to massive subsidy payments have coined “public money for public goods” describing the public perception that industrial agribusiness had become the main beneficiary of the CAP. This reform has not been adopted in the United States [21,41]. The Environmental Work Group maintains an updated database and analysis of the expenditures of the Farm Bill subsidy policy in the United States [42].

Many critics agree the US Agri-industry has been farming the mailbox since the introduction of the Agricultural Adjustment Administration 1933 and has developed a heavily fortified government lobbying and political presence that will prevent any infringement on its almost century long hegemony. Implementing agro-environment and conservation programs into the Farm Bill have always been low cost window dressing compared to the subsidy component (surplus buy-up, lunch and food programs, direct payment to “farmers”). Changes in method of production for animals and oversight of animal use in the agro-industrial complex will not be met with other than open hostility. However the Farm Bill and most of agriculture policy instruments in Canada have been directed at crop production. In Canada and the USA agricultural farm gate sales are approximately 50% crops and 50% livestock and livestock products [43], so the concentration of subsidy on crops is not due to their predominant value in the market but due to historical corporatist power arrangements [44]. In the perpetual U.S. Farm Bill, a few commodities, for example, sugar and dairy, are highly protected by tariffs and import restrictions. Another group of commodities (wheat, corn and other feed grains, soybeans and other oilseeds, rice and cotton) receive most of the subsidy payments. A third group of commodities (fruits and vegetables, livestock and poultry) receive little direct support, so innovation in livestock does not come into direct conflict with entrenched federal ‘acre/bushel based’ farm policy [19]. 

### 3.4. The Problem of Path Dependency

Path dependency theory originated in the field of economics to explain why seemingly inefficient technologies succeeded and sometimes monopolized markets despite better alternative technologies (e.g., VHS videotape format captured the market from Betamax). Path dependence is a perspective within political science in those initial decisions and conditions almost irreversibly affect the subsequent breadth of possible future decisions. For instance once an imperialist government has decided to implement a reservation system for previously indigenous peoples that decision (and all its unanticipated ramifications) is largely irreversible and all future policy options are greatly restricted. Over time, social scientists, in particular those interested in political institutions and policymaking, have begun to use path dependent approaches in their analyses. The decisions that are significant and irreversible are called “choice-points”. The decisions made by President JF Kennedy during the Cuban Missile Crisis (1962) were taken with great care as this was an easily recognizable choice-point in the theory of path dependency. 

Path dependency suggests that once a society, or in this discussion public policy, has started down a track, the costs of reversal, or adoption of an alternate approach are very high. In addition, once a decision is made, especially in an area of high potential profitability, government bureaucrats and free market agencies proliferate whose survival depends on, the government never entertaining the possibility that a policy error could have been made. The dependent institutions and agencies lobby violently for maintenance of the status quo. This is a major factor in resistance to any change or reform in health care [45,46] Once an institution has been established, it gains a permanent place among the tools available to society; it persists, finds ways to challenge opposition and suppress criticism and can be adapted to new political purposes [47]. In the United States, the majority of agricultural policy research is funded by the United States Department of Agriculture (USDA). Some economists believe this arrangement creates a perverse incentive for inefficient historic government intervention to persist because, among other considerations, the USDA will most likely not fund or publish research criticizing its own activities [48].

There will be other choice points, but the entrenchments of certain institutional arrangements obstruct an easy reversal of the initial choice [49]. Current society-government-industry institutional arrangements will resist change and if future new intuitional arrangements are hard won, they in turn will also resist change [21]. This historical reality is instructive in that society must choose wisely when choosing an approach to correct the current deficiency in social responsibility in the area of farm animal welfare assurance.

Path dependency theory regarding animal welfare law enforcement would suggest that the construction of the charter of the American Society for the Prevention of Cruelty to Animals (ASPCA) could have been or was an accident of time. The charismatic personality of Henry Bergh, combined with the evangelical righteousness of the Elizabethan morality in high societal circles in New York, the relative weakness and novelty of uniformed police services and the hubris of the New York State Legislature all combined to achieve a Nation-wide charter for the ASPCA. The ASPCA purpose, as set forth in its constitution, was “[t]o provide effective means for the prevention of cruelty to animals throughout the United States, to enforce all laws which are now or may hereafter be enacted for the protection of animals and to secure, by lawful means, the arrest and conviction of all persons violating such laws.” granted on April 10, 1866. Henry Bergh was unanimously elected as the ASPCA's first president, a position he continued to hold until his death in 1888 [50].

The survival of the ASPCA may have more to do with an early mutually beneficial partnering with media as accomplice in fund raising activities [51] to save the animals and provide social support for the organization. The media assists in raising the profile of companion animal “rescue”, and high profile vilification of animal abusers functions to drive the fund raising efforts of police empowered animal rescue organizations. Currently the media is a participant in creating and maintaining terrorism as a form of political theatre to maintain needed political support for military intervention in American interests overseas [52].

### 3.5. Policing Farm Animal Production—Theoretical Constructs

Sklansky [53] eloquently articulates the difference in organizational cultures between a “Governance Organization” such as the RCMP-GRC (Mounties in Canada) which is a non-unionized professional police force and a “Managerial Organization” such as Blackwater International [54]. Governance Organizations operationally take into account broad social values such as individual officer integrity, the fair accommodation and respect for all interests, local social norms, and societal morality. A “Managerial Organization” operates to express values of efficiency, achievement of targets and pursue very narrowly defined private goals such as the example of the Pinkerton National Detective Agency [55]. Churchill’s extensive review of the history of Pinkerton agency and its aggressive and violent “for profit” suppression of trade unionist movement in the United States is a sobering reminder of the risk of private policing circumventing constitutional protection of civil society. He also makes a convincing argument that significant traces of the managerial culture of the original Pinkerton agency of, results at all (social) cost, has penetrated and been maintained in the current operations of the Federal Bureau of Investigation [55]. Private Military and Security Companies (PMSCs) in Iraq and Afghanistan demonstrated significant loss of governance and transparency and particular difficulty in detecting extraterritorial transgressions and crimes against civilians. Subsequent to a specific incident in Iraq, Blackwater International a PMSC, closed business and reappeared under a new name Xe International [56].

In current academic police studies, police organizations are classified as functionalist, divided society or predatory. In states with the functionalist model of policing, the police are a public service provided to enforce the law, protect the public and preserve order increasing the general public good. Social surveys will report that less than 1% of the citizenry have experienced police corruption or violence in societies with a functionalist police culture. 

In divided society policing, the police largely act as an arm of the central government to suppress minorities and dissidents and may provide some effort to solve or prevent crime. In divided society policing there is a systematic discrimination of police against subordinate groups, strong identification of police with the regime in power, and polarized communal relations with police. The subordinate group is estranged from the police and distrustful of the legal system as currently often the challenge to policing in marginalized ethnic communities in inner cities. Divided society policing is manifest most dramatic in the “police state”; the first being Prussia under Frederick William I (1688–1740) [57]. The early French police Renseignements Généraux, also had a strong mandate to collect information about private citizens and a strong political homage to the regime in power, both distinctive components of divided society policing.

Predatory policing is a relatively new concept to describe certain organizational problems in developing democracies in the wake of totalitarian regimes, or release from colonial control. In predatory policing, the police do not serve a political purpose as there may be few minorities or political dissent; however, the police are usually very poorly paid and systematically use intimidation, violence and force to collect rents from the general public with little fear of internal or external reprisal. Social surveys of citizens experiencing a culture of predatory policing report that more than 10% of the citizenry have experienced police corruption such as bribe seeking or violence within the recent past. Corruption is expected from the uniformed police and all citizens generally avoid the police and distrust the legal system [58]. 

The livestock farming community is an exquisitely small fraction of the general public. Policing this group, due to its extreme minority status is at risk for both divided society and predatory policing depending on the organization of the policing agency.

#### 3.5.1. Paying for Policing

Some government services perceived as a general public good can be fully funded from the tax base, such as a minimal level of public education. If other government services with a component of private benefit are free, then there is no feedback control on the volume of demand and social disutility can result. However, there are three broad ways in which government services can be partially privatized and funded. The most just method of offsetting the cost of a shared benefit is that each beneficiary pays in proportion to their individual benefit (or ability to pay). Where a general good such as clean drinking water, or security of the person, is 100% user pay, essential services may only be available to the rich resulting in social injustice. Few social services are deemed essential. Many government services are privatized to a certain extent, most commonly represented by a user fee, but the government retains the sole delivery agent such as production of a national passport. With some animal welfare policing provided previously provided without state subsidy, cost sensitive governments will attempt to retain as much of that system as possible in the future. 

If both the funding mechanism and the service delivery are shifted to the private sector we have the most complete form of privatization referred to as load shedding [59,60]. Privateering where a National Government act to commission civilian naval vessels to arm themselves and participate in international aggression is an old and established load-shedding practice in Anglo-American international disputes [61].

In North America the most dramatic example of recent load shedding is contained in the action of the British Empire in giving force of government to The Hudson’s Bay Company Charter (HBC) a private, for profit, Managerial Organization. For 200 years (1660–1869) the HBC was the de facto government of what are now the provinces of Manitoba, Saskatchewan and Alberta and parts of the Northwest Territories and Nunavut [62]. It was at that time the largest landowner in the world, with Rupert's Land containing 15% of North American land area. Unlike modern democratic sovereign states, the Managerial Organization of the HBC made no pretense that general security of the person would be a priority or be extended through the vast range of territory held by the company [63]. The HBC territory was essentially a society without civil policing and with no distinction between Crown Corporation and Government. 

Most political science authors suggest that security of the person is a prerequisite for the exercise of civil liberties in liberal democratic societies [64], and is in fact proof of the existence of a legitimate government. There are a significant number of sovereign states where there concurrently is an absence of civil liberties and concurrent lack of individual security [65].

#### 3.5.2. Self Funded Policing: The Christian Inquisition, the ASPCA Model and the War on Drugs

These three historical social engineering movements share four distinct characteristics. They were dispersed social control activities; were initiated with a limited investment in infrastructure; they expanded and persisted for significant periods of time, over 400 years for the inquisition; and that they followed a business model of predatory policing. 

##### 3.5.2.1. The Christian Inquisition (1231)—Load Shedding

The Inquisition (inquisition heretice pravitatis, inquisition of heretical depravity, IHP) functioned much like the secret service of any totalitarian government [66]. The IHP was implemented by a group of specialist decentralized actors/institutions within the justice system of the Roman Catholic Church whose aim was to "fight against heretics". In the 11th and 12th century’s geo-political complexity of Christian Europe resulted in ongoing schism and the emergence of new orders within the Catholic Church [67]. These new, usually evangelical divisions (orders) had to be either given a job and retained within the church umbrella or denounced and stamped out. The appearance of the Mendicant Orders in the early decades of the 13th century, first the Dominicans and later the Franciscans took it upon themselves to fight heresy, to urge Christian mission among the infidels, and to convert the Jews (Iberian Peninsula) [68]. The formalization of the IHP as a medieval institution is generally dated to 1231 when Pope Gregory IX announced his Papal Bull, Excommunicamus [68]. The Excommunicamus established inquisitorial courts answerable directly to the Pope [66]. These courts bypassed courts created by local Bishops, as had been Church practice until the thirteenth century [69]. These instrumental functions of the Mendicant Orders, as primarily men of action not letters became useful to the Church. The IHP started in earnest in 12th century France to successfully combat, suppress and eliminate the heresy of the Abilgenses, a specific religious sect and regional (Dominican) inquisitors were later appointed as prudent in other European countries.

Most writing on various geographic inquisitions focuses on excessive use of force, torture, and the obvious conflicts of interest and corruption in the system. For the purposes of this discussion, it is the business model of investigation and policing that is of interest. No loosely bound organization can recruit staff, persist for hundreds of years, spread geographically and multiply without a sound business model. *Sequere pecuniam* (follow the money) is a useful way of understanding how the load shedding aspects of the inquisition business practice invariably led to excesses and abuse. What inquisitors (whether Dominican or Franciscan) had in common, was a bureaucratic strategy and legal mechanism. As church appointed officers they had wide discretion to cooperate with the local secular powers to bring people to trial, convict them and then hand them back to the local authorities for punishment. Critical to punishment was forfeiture of all chattels, property and proceeds. Although forfeiture and confiscation was officially a secular activity, a holdover of prior Roman Law, only in France did the secular ruler retain 100% of proceeds of conviction; the actual division of proceeds between the church and local politicians varied by time and place [70,71].

This provision of police power without oversight established is a rigorous business model where the proceeds of confiscation assure resources for future prosecutions. Especially profitable was IHP prosecution of the memory of the dead which could occur up to 100 years after the person’s death. Defense was virtually impossible in prosecution of the dead and confiscation of all property and chattels of the descendants was assured by the taint of heresy. An excellent review of the extent of the prostitution of religion in the service of greed in Spain is given by Henry Charles Lea [70]. In Spain the prosecution and conviction of the (wealthy), primarily Jews and Conversos (recently converted Jews), with forfeiture of all property, unremittingly for three centuries was a tremendous burden on the productivity of the most industrious segment of the population. As the orthodoxy secured hegemony in a region no personal property, business partnership or the life of any person was ever assured. Any business transaction or joint held property could be reversed or lost at any time by the retroactive forfeiture powers of the inquisition. It is no surprise that the Netherlands, Germany and England where Protestantism emerged were also witness to the development of modern banking, business and joint capital investment which allowed for business to develop venture companies and soon after financed empires [71]. 

##### 3.5.2.2. The Formation of the ASPCA (1867)—Load Shedding

Public policing is a relatively recent development in democracies [72]. The first public police department in New York City was established in the 1840s [50]. In the 1867 amendments to the New York State Penal Law, one of the earliest significant animal protection laws in North America, the American Society for the Prevention of Cruelty to Animals (ASPCA) was given private police powers [50,73]. This delegation of state criminal authority to a private organization was a unique and extraordinary approach. 

The establishment of the ASPCA in New York in the 1800s was an example of load shedding where, by design, enforcement of animal welfare inspection and prosecution was intended to be funded by the fines received from successful prosecution. In application, however, the newspapers generated high visibility for prosecutions which were co-marketed with voluntary donation drives. The voluntary donation and bequest became the basis for financial support of the organization. Income from fines was never a significant contributor to any SPCA operating revenue [50]. The “public spectacle of sentencing” in animal welfare prosecution and the public execution of heretics functioned in a similar way to communicate a message to the public [74]. High visibility animal welfare spectacles such as Michael Vick’s arrest and conviction [75] have “spectacle” qualities of the public hanging or burning at the stake [76]. 

The Ontario Society for the Prevention of Cruelty (O-SPCA, ON, Canada) was founded in 1873 (6 years after the ASPCA) to operate shelters to assure welfare of animals and children in the Canadian province of the Ontario. The safety of children was later transferred to a parallel organization the Ontario Children’s Aid Society. The policing of animal protection was added to the role of local police officers (uniformed police services, UPS) in the province in 1887 [77]. In 1955, in a remarkable divergence from all other provincial statutes, the O-SPCA Act was amended to displace the local UPS role in investigating complaints, with special inspectors from the O-SPCA [78]. In a remarkable example of the “policing family” not playing together well, the O-SPCA Act actually prohibits uniformed police officers from investigating animal abuse unless the region is so remote that the O-SPCA inspectors are unable to service it [78]. In addition the O-SPCA holds legal control within the province over the use of the phrases “humane society”, “society for the prevention of cruelty to animals” or “spca” or the equivalent of any of those names in any other language, alone or in combination with any other word, name, initial or description (Paragraph 10(1)(b)) [78]. In this business model, there is competition between animal protection agencies for limited voluntary donations and for public visibility. In similarity with the organization of the Christian Inquisition, where power is concentrated one would anticipate that the competition, other organizations in the animal shelter business (heresy) must be dealt with.

This extreme example of load shedding of social control was recently critically reviewed in an Ontario Court of Justice decision, R. v. Pauliuk, 2005 ONCJ 119 [79]. In the ruling regarding the rightful seizure of horses, the Judge indicated the full police powers of the O-SPCA are executed by the organization, all the while attending to its own need to fund raise. In order to raise funds, the O-SPCA relies heavily on the publicity it can glean from high profile seizures with or without eventual prosecution. The O-SPCA maintains a communications branch; tasked with fund raising, in part, by maximizing the public visibility of enforcement activities. In reviewing this organizational arrangement, the Honorable Mr. Justice Zuraw J wrote; *Without publicity and high profile charges, the funds the OSPCA needs to operate would no doubt dry up.* This quote bears a striking resemblance to a reference to the 1208 Pope Innocent III and Philip Augustus, King of France attempt to eradicate the political-religious group in southern France the Albigensians. *The money and property of convicted heretics were confiscated by the Inquisition. In fact, the organization was dependent on the revenue from such confiscations for its survival* [80]. In looking for the next “poster puppy” the O-SPCA may be acting as a predatory policing organization.

##### 3.5.2.3. The War on Drugs—Proceeds of Crime Law

The best-documented moral hazard associated with load shedding is in the administration of the forfeiture of proceeds of drug trafficking in the United States [60,81,82]. The law enforcement agenda in the drug war has shifted to target assets (recover money) and not prevent crime [83]. For example, in the administration of state and federal forfeiture powers, it is more profitable for enforcement agencies to delay intervention until drugs are sold illegally and then to seize the cash, than to seize the contraband, which must be destroyed [84,85]. The seized cash, cars, personnel effects, owned real estate as proceeds of crime can be divided between participants (police, judiciary, Prosecutor) similar to the division of property of convicted heretics 500 years ago. 

The actual outcome of over 20 years of “the War on Drugs” is drugs are more available, at higher purity and lower prices, than they were at the start of the “war” [84]. Much like the business model of the Inquisition, the drug war forfeiture powers, especially the development of Civil Asset forfeiture, where no criminal conviction is necessary has achieved independent, self-funding and self-perpetuating police companies [86].

Civil Asset forfeiture as practiced in the US is very attractive. The property itself is charged with the crime and property is not protected by any constitutional restrictions which would be available to the owner if charged criminally [86,87]. In addition, like prosecution of the dead for heresy, civil forfeiture law in the U.S.A. allows for proceedings against the property of the dead [87].

Operating a police force based on the proceeds of crime or seizure of real property with the taint of crime may initially appear immoral as an articulated public policy [88]; it is however fully rational as a political and bureaucratic strategy. The forfeiture laws in particular are producing self-financing, unaccountable law enforcement agencies divorced from any meaningful legislative oversight [82]. There are numerous examples of such semi-independent agencies targeting assets with no regard for the rights, safety, or even lives of the suspects [84]. This process is a near replica of the forfeiture provisions available to the prosecutors of heresy, which was a very resilient and durable paradigm; one that required the emergence of protestant states to challenge.

These examples of load shedding are relevant to any regional attempt to modernize animal protection law and extend it to farm animals due to the pernicious effects of path dependency. In a review of the development of new institutions to protect farm animals Sankoff [89] describes the evolution of the enforcement complex in New Zealand. With the new 2000 law a branch of the Ministry of Agriculture (MAF) was empowered to prosecute in the enormously expanded area of regulated activities alongside the Society for Prevention of Cruelty to animals (SPCA). These completely different organizations have widely different cultures, structure and source of funding, a “legacy”. The SPCA is left the option to prosecute that MAF deems lower priority or not in the public interest to follow up. As organizations competing for public lauding they do not share resources, legal knowledge and both entities retain full organizational independence and jealously guard’s information. As in other countries, the SPCA is self funded through donations and memberships; tasked with lobbying the government on behalf of the citizen concerns, and in preventing the death of stray and surplus animals where possible. 

### 3.6. Legitimacy—Procedural Justice Model

Research over the past decade has repeatedly demonstrated that fear of punishment or hope of reward does not factor into individual compliance with the law [90,91]. Most people comply with the law because they believe the law to be just and necessary and compliance is the right thing to do. In functional police practices the police use little force and seldom sanction, and police with the consent of the regulated [92]. In a well functioning policing situation the law, police as an organization, and the individual officer all have “legitimacy” in the community targeted for police services [93]. Policing using the procedural justice framework is grounded in empirical research that demonstrates individual compliance with the law, and willingness to cooperate with enforcement efforts are not shaped by the threat of force or the fear of consequences. People are law abiding because of their belief the law is just and law enforcement agencies are legitimate. Police are viewed as legitimate where their behaviour displays the attributes of procedural justice; that policies are formulated and applied fairly, so that regardless of material outcomes, people believe they have been treated respectfully and without discrimination [94,95]. 

Legitimacy is a judgment people make about the status of an organization itself and is an internalized belief that an authority does their job well and is therefore entitled to be obeyed. Legitimacy also reflects the level of confidence and trust people have in those authorities. Where authorities are judged to be legitimate, people feel that they ought to defer to their decisions and rules, cooperate with them and follow them voluntarily out of obligation rather than out of fear of punishment or anticipation of reward [94]. An authority itself may be seen to have legitimate authority, but people can still call into question the legitimacy of the policies, rules and laws that those authorities are enforcing. In such a situation, compliance or voluntary cooperation with authorities may be less likely [93]. Central to the individuals to which the law applies, legitimacy of social control systems turn on two ultimately interdependent issues: (1) the legality of the activities of law enforcement officials, and (2) whether and to what extent the law itself and the manner of its enforcement express the “shared values” of the community within which that law operates [96].

There is substantial empirical evidence to show the importance of legitimacy in achieving law-abiding behavior and cooperation from citizens, especially through what has been described as procedural justice; that is, quality of decision making procedures and fairness in the way citizens are personally treated by law enforcement officials. Procedural justice concerns the perceived fairness of the procedures involved in decision-making and the perceived treatment one receives from the decision-maker. In other words, it relates to how a person may perceive the interpersonal treatment they have received from an authority, regardless of whether the resulting outcome will be favorable or not. Research into the effects of procedural justice has consistently found that people and organizations are much more likely to obey the law and accept decisions made by authorities when they feel that the decision-making procedures are fair, respectful, and impartial [92,97]. They are also more likely to report wrongdoing to an authority that has treated them fairly [93].

Max Weber argued that legitimacy in this internalized social sense was key to the effectiveness of the state [98]. Cooperation is a more valuable and more fragile commodity than compliance, because there is usually no recourse to non-cooperation. Zealous animal protection agencies tend to operate under an instrumental paradigm of a “crime control model” (one that identifies offenders and delivers just retribution). Under a functional model of animal protection with the emphasis on a “due process model” the priority is one that focuses on preventing animal injury, maintaining respect for individual rights, recognition of pluralism in society, and the value of human decency. 

Applying the legitimacy theory of social control a functional animal protection system is not about war, or identifying and naming and shaming criminality; it is about working toward social compliance. This attitude can clash with individuals solely motivated by a compulsion to “save the animals” and punish the offender. The key concept in enforcement by social and psychological legitimacy, is trying to understand the view from the perspective of the target audience whose compliance is sought, in our theoretical case, farmers and farm workers. 

Studies in both the civil and criminal field of “procedural justice” have demonstrated that there are three factors that determine whether or not an individual believes that a given procedure is fair. The first is known as “process control”, which is the individual's opportunity to participate in the procedure, whether or not their participation affects the actual outcome. The second factor is whether the participant views the decision maker as neutral and unbiased—that is, whether the rules are impartially followed and the decision maker appears motivated to be fair to both sides in a given case. The final consideration is whether the individual is treated with dignity and respect during the process [90,99]. 

The current O-SPCA model of self-funded, vengeance seeking, righteous evangelists for animal rights cannot attain any level of the principals of procedural justice and will not be accepted as legitimate by the livestock producer.

## 4. Agriculture and the Constitutional Division of Powers

In dual-federations such as Canada and the USA both the National (Federal) and Sub-national (Province, State) independently make laws and collect tax revenue. Many scholars writing in animal law assume that animal protection will remain in the umbrella of criminal law [100,101]. The standard goals of the criminal justice system are (a) retribution against those who commit crimes; (b) incapacitation of offenders so that they cannot: commit more crimes, escalate criminal behavior, or the corruption of others in the near future; (c) general deterrence by showing other individuals engaged in anti-social and criminal activity the potential outcome of their career choices (prevention); and to a lesser extent (d) rehabilitation. These primary “public goods” are commonly referred to as retribution, incapacitation and general deterrence [99]. One of the additional purposes of a criminal law is instructional, to inform the citizenry of an agreed to behavioral norm. This author asserts that due to the long term social acceptance of farming practices and their invisibility to the general public criminal law has no possible utility in providing increased oversight of livestock production systems.

The criminal justice system attempts to deliver “justice”, after an offence has occurred, primarily with inherently bad people displaying serious anti-social behavior. Animal protection law is quazi-criminal, in that the regulated may or may not be aware that their actions are out of alignment with societal norms. In addition, the purpose of animal protection legislation is usually to prevent animal injury not to punish the offenders. In agriculture under some future regulatory oversight, compliance officers will generally be working with otherwise law abiding, respectable citizens (good people), who have inherited and adopted socially unacceptable norms related to animal use. No government will create a new class of felons overnight and any successful oversight of farm production must come out of administrative law, and develop as policing-by-consent, especially in dealing with the corporate nature of livestock production [102,103].

### 4.1. Federalism and Animal Protection Law

Intergovernmental arrangements (IGAs) are official and unofficial organizations which are set up by national and sub-national actors within distinct types of polities. It has been convincingly shown that the density of these exchanges has increased across Western federal systems [104,105]. Most striking, however, is that in some countries such as Canada, cross-boundary exchanges are still primarily channeled directly by the respective National or Provincial ministries. Canada is characterized by a governance system of voluntary mutual adjustment (ad hoc coordination), which does not demand any of: regular meetings, a bureaucratically supported and internally differentiated body, a formal decision making rule, or the legally binding status of agreements as is characteristic of Cooperative Federalism. 

As an example of dual-federalism, Canadian First Ministers meetings [Federal Prime Minister (PM) and provincial Premiers] are called irregularly and at the lone prerogative of the PM. There is no schedule, no standard agenda, agenda items are at the discretion of the PM, and typically federal-provincial meetings are called when the federal party in power will stand to gain the most. Each sub-state (Province or Government of Canada) possesses considerable law-making authority as well as its own taxing powers, and hence can withdraw from interaction and resort to unilateralism whenever it considers such a path politically profitable. Canadian Provinces and the National Government cannot hold each other accountable to agreements and no constitutional process are available to determine agreement development. In addition both levels of government are represented independently to the Crown (British Monarch) [106]. 

A good example of failure of dual federalism in Canada is in the 25 plus year initiative to create a National Child Care Program. After many previous attempts the Federal Government and provinces came very close to an agreement in 2005, which was abruptly ended by a unilateral cost cutting initiative of the federal government [107]. Highly complex and integrated social programs are difficult to communicate to the public and difficult to sloganize. Issues with a high level of citizen participation like universal child care are often vetoed on simplistic ideological grounds rather than cost benefit or otherwise carefully considered good government decisions [108]. 

As an example, in 1971, then US President Richard Nixon vetoed a $2.1 billion Bill to provide comprehensive child care. His veto message spoke of the threat of ‘Sovietizing’ the American family: “*For the federal government to plunge headlong financially into supporting child development would commit the vast moral authority of the national government to the side of communal approaches to child rearing against the family-centered approach*” [109]. Over 30 years later, the 2005 message from Canadian Prime Minister Stephen Harper; had previously criticized the calls for a national child care policy by saying, “*There already are millions of child care experts in this country. Their names are mom and dad.*” [110]. Sabotage of expensive and complicated previous intergovernmental negotiations (as a farm animal welfare agreement would be) is a particularly easy and high visibility election promise to keep.

As Canada is an extreme example of dual-federalism, the federation of Germany is the prototype model of cooperative federalism. Germany demonstrates the empirical features of cooperative federalism with systematic obligatory National-Subnational government meetings, a bureaucratically supported and internally differentiated body outside of a single level of government, a formal decision making rule, and the legally binding status of agreements, which are all characteristic of a highly institutionalized environment facilitating National-Subnational co-decision [104,105]. Cooperative federations by design have strong institutions, with a strategic agenda which allows the country to survive and minimize the highly partisan damage of periodic elections in dual federation democracies. A core feature of strong institutionalization is a formal decision-making rule and process that empowers the institution (differentiated body) with the capacity to bind the Federal and the State participants to common positions or plans in the absence of unanimity. 

Federal constitutions where more powers are assigned to closed “watertight compartments”, the weaker the incentives for cross boundary interaction. The more the federal constitution provides for wide areas of concurrent powers, the stronger the incentives to develop strong intergovernmental relations and cooperative decision making. Looking at the areas of concurrent (seamless) policy and legislation, based on a range of policy areas, Germany has 62 percent of concurrent Federal-State legislation-policy arenas, whereas Canada has only 2.5 percent common interest (Federal-Provincial) and the United States 13.6 percent [111]. Canada and the USA are both considered dual federal systems in contrast to cooperative ones. In both Canada and the USA there are a large number of constitutional exclusively assigned competencies. Exclusive areas of power allow the two orders of government considerable autonomy; to exercise their respective powers without consultation or agreement from the other level of government [105]. However, in both countries, agriculture is more of a shared responsibility than many other policy areas. 

As an example of a differentiated body outside of a single level of government, the USAHA (The United States Animal Health Association) is an external organization that facilitates consensus on animal health policy between the federal government represented by the USDA (United States Department of Agriculture) and the State Veterinarians. There is no equivalent veterinary policy organization in Canada. In fact, the most recent discussions on the joint delivery of a national livestock program (an animal health program) was on February 20–21, 1950, Ottawa, to design the national cost shared program for the vaccination of cattle for the prevention and control of brucellosis [112]. Since the sunset of the “Federal-Provincial Brucellosis Control Program” there has been essentially no success or significant effort to develop national Federal-Provincial programs related to livestock production in Canada. In contrast there is a strong history of Federal-State delivery of animal disease eradication programs in bovine tuberculosis, bovine brucella and most recently Pseudorabies of swine [113]. A small non-profit organization (Canadian Animal Health Coalition) has supported the production of a document “A National Farm Animal Welfare System for Canada” [114] which documents the lack of any mechanism to create national farm animal welfare policy in Canada.

### 4.2. Example of Dual Federalism Fail: The California Downer Cow Law

On February 17, 2008, USDA announced that Westland/Hallmark Meat Co. of Chino, California, was voluntarily recalling 143.4 million pounds of fresh and frozen beef products dating to February 1, 2006. At least 50 million pounds were distributed to the school lunch and several other federal nutrition programs. This, the largest U.S. meat or poultry recall ever, came after evidence that this facility had a practice of occasionally allowing the slaughter of cattle that had become nonambulatory after they had been inspected, but before they were slaughtered for human food. The Food Safety Inspection System (USDA-FSIS) regulations explicitly prohibit nonambulatory (“downer”) cattle to be slaughtered for human food, because they are more likely to have bovine spongiform encephalopathy (BSE, or “mad cow disease”). It was this remote disease hazard that empowered the USDA to trigger the meat recall that bankrupted the company [115].

The real public outrage was directed toward the apparent inhumane handling of downer cows not any theoretical disease risk. This incident in California initiated a series of events which resulted in a California state regulation prohibiting the processing of all non-ambulatory animals for food and required the immediate humane killing of such animals [116]. 

Under the Supremacy Clause of the U.S. Constitution, federal law may preempt state law, either expressly or by implication. The US Supreme Court struck down the California downer law, which had amended the California penal Code Sec. 599f, (National Meat Assn, *vs.* Harris 2011) [117]. In a unanimous decision authored by Justice Elena Kagan, the Supreme Court reversed the lower court, holding that the Federal Meat Inspection Act's preemption clause (FMIA, 21 U. S. C. §60) applied broadly to any additional or different regulations a state imposes on slaughterhouses. The FMIA prevails even where state law does not conflict with the federal act. The Court rejected arguments that the Act did not apply to animals rendered non-ambulatory before reaching the slaughterhouse. The Court also held that the criminal penalties imposed by the state law were more than a mere incentive to improve humane animal slaughter practices. We can anticipate a future of intergovernmental legislative wrangling as the political and jurisdictional stakes are considerable in dealing with the legislative control of agriculture production methods.

## 5. Obstacles to Improving Farm Animal Welfare

There are a plethora of obstacles to the implementation of regulatory oversight in the livestock industries in North America. This section will consider only the major easily identified classes of problematic areas. At present, the idea of regulatory oversight of method of production of farm animals is a paradigmatic rather than an incremental change and this magnitude of change can only happen when the policy system is open to new ideas and influences [22]. 

### 5.1. The Problem of Scale

If we consider that current animal protection laws primarily govern the way companion animals and horses are treated; that number is a small fraction of the total animals used by man and is in the order of millions. The livestock industry consumes billions of animals annually [118,119]. In addition the regulation of agrifood systems has shifted. Whereas nation-states used to be the primary regulators of agrifood systems, the new agrifood regulatory terrain now includes, not only nation-states, but also global governance organizations (e.g., WTO, OIE), multilateral and regional regulatory schemes (e.g., the European Union), and private sector organizations, including transnational corporations (e.g., Cargill, McDonalds and Wal-Mart) [120]. 

### 5.2. Complexity—Lack of a Bright Line

A bright-line rule generally used in law (or bright-line test) is a clearly defined rule or standard, composed of objective factors. The opposite is a fine line rule in law where not every judge can agree where the actual line is. A bright-line rule is made in Anglo-American common law usually by a Supreme Court ruling on a previously fuzzy legal concept [121]. In many animal protection statutes there is wording like “undue suffering”, communicating that some animal suffering is legal and undue is based on a balance of interests. In writing new regulations, there is an attempt to make them as “bright-line” as possible. Where the law is clear, it leaves little or no room for varying interpretation. The purpose of a bright-line rule is to produce predictable and consistent results in the application of law. “It is an offence to exceed the posted speed limit” is a clearly articulated bright-line law, where “dangerous driving” is a vague description of a human behavior which the law (society) intends to suppress by making it a series of bright line offences (licensing, blood alcohol levels). 

Livestock production is a very complicated science based process and the lines are difficult to draw and are almost always vague. The offence “failure to provide adequate drinking water” is a fine line rule. Is the offence proven when an animal becomes thirsty, looks for the water bowl, pushes over a fence to get to water, or not until the animal has died of dehydration? 

Describing laws, especially what is a “violation” can be exceedingly difficult. For example The 10 Commandments contain 297 words; The Bill of Rights (USA) is stated in 463 words and the Bovine Spongiform Encephalopathy Minimal-Risk Regions and Importation of Commodities; Final Rule and Notice Federal Register: January 4, 2005 (Volume 70, Number 2) contains 107,648 words. 

Many discussions on farm animal welfare regulation have focused on the lack of objective science (bright line) to clearly demonstrate that one method of production is superior to another method [122,123]. The expectation being, that when science delivers the bright-line rule, then the competent authority can enforce it and the industry will naturally adopt it. The focus on the science basis for animal welfare standards may in fact be missing the yet unresolved point. Regulation is not based only on science but on moral motivation to protect human welfare, and respect the intentions of the electorate [124]. Devising a regulatory framework to get better welfare outcomes for production animals is extremely difficult [125]. It is very difficult to write a good animal welfare law. Thin line rules may be all that is currently available to farm animal regulators, difficult to convict on and not supported by the regulated. 

### 5.3. War on Farming

Introduction of new animal care legislation primarily at the sub-national level is an indication that the status of animals, particularly “companion animals” is changing, as society ponders new evidence regarding the sentience of animals and their place in a human world [126]. Most animal care laws offer as a primary legislative objective the establishment of a culture of respect for animal welfare and the recognition of the symbiotic relationship between animal and human welfare [9]. Legislation is always piecemeal and goes only as far as is needed to take the pressure off of the legislators. In highly contentious issues there will be some science on both sides of the argument and the final policy decision will be based on ethics and/or political expediency [124]. 

Military/criminal styles of policing are based on actions taken post injury [127]. The crime must happen, the victim must suffer, then the police will engage to identify and punish the perpetrator. In theory, enthusiastic punishment deters others from committing the crime. The broken-window theory of policing, where every social deviance no matter how small is speedily crushed, is very expensive to the policing authority and to the community subjected to the Crusade like conditions. In addition, the police constantly acting in a threatening way maximize their alienation from the community. 

If democracy is understood to be primarily a method of guarding society from unjustifiable tyranny then the public police are both a uniquely powerful weapon against private systems of domination such as organized crime and a uniquely frightening tool of official domination as demonstrated by the East German Stasi (*Staatssicherheit*) [54]. Farm organizations are very cognizant of the urban nature and potentially anti-farm bias of current self-funded animal welfare enforcement organizations.

The physical restraint of a 600 pound calf (300 kg) in a head gate and the use of scoop dehorners, where the horn base and a sizeable chunk of the skull is removed without any pain control [128] should be considered barbaric in any cultural context; yet, it is a common livestock management practice, exempt from most regulatory processes in North America. Achieving substantial shift in farm animal production methods means a coordinated societal initiative to disseminate and have adopted a new societal norm. Criminalization of a current behavior, considered normal by a significant segment of society may not be the most effective method of getting the outcomes for animals we want. Based on the failure of the “War on Drugs”, and experiences of policing in foreign cultures such as Afghanistan and Iraq, the use of blunt force to achieve compliance may not be economically possible and a new way of winning farmers’ hearts and minds may be required. 

With the current populist support for neo-liberal conformity (all government intervention is evil) it is likely that a clear demonstrated failure of all market based animal welfare programs would be a prerequisite for significant state regulatory action in North America. North America is considerably behind the EU in experimenting with method of production certification, and on farm welfare assurance. 

### 5.4. Regulatory Capture

Regulatory Capture is a term identifying situations where the regulators work very closely with the regulated intending to assist the audience to comply, and over time become subservient to the industry they are tasked to oversee; the outcome being counter to the societal interest for which the law was originally designed [129]. The study of regulatory capture is most frequently in the area of economics and is the process by which regulated monopolies end up manipulating the state agencies that are suppose to control them [130]. Regulatory capture has been identified as a primary contributor to the sub-prime mortgage financial crisis of 2007–2009 [131,132,133]. Although most economic reviews of this recent economic disaster call for increasing objective and competent regulatory oversight of Wall Street finances, the neo-conservative dogma of Reagan-Thatcherism is so pervasive that creative authors can still manage to blame the government regulators for the recent financial crisis [134].

In Regulatory Capture, civil society loses control over the policing authority because the authority becomes identified with the regulated industry and facilitates the goals of the industry. In assuring farm animal welfare, if preventing abuse is the goal, then some form community policing is the preferred option. However it has not been demonstrated that an internal system can foster a significant change in the social norm of livestock workers, by education, influence and cooperation without the negative aspects of capture [135]. 

The regulatory approach of incremental animal welfare improvements [123] requires mentoring by a legitimate then current trusted policing infrastructure strictly adhering to a procedural justice model. Embedded institutions such as state departments of agriculture or the sub-national veterinary infrastructure are candidates for community policing driving incremental improvement. In the USA where there a perception of society’s need and the possibility of a profit self funding organizations will emerge for example Professional Animal Auditor Certification Organization, Inc. [136]. This organization formed in 2004, made up of a coalition of five professional animal industry organizations, none of which represent animal welfare or protection as a primary mission, may have limited credibility as an arm of social change, and appears to function without the inclusion of civil society.

The clearest example of regulatory capture in the area of animal welfare is manifest in the longstanding experiment of the USDA in load shedding the responsibility to deliver The Horse Protection Act (HPA), passed by Congress in 1970. The HPA (PL 91-540) prohibits the showing, sale, auction, exhibition, or transport of sored (intentional injury to the foot) horses. Congress found and declared that the soring of horses (single breed—Tennessee walking horse) is cruel and inhumane, and that sored horses, when shown or exhibited, compete unfairly with horses that are not sore. Congress amended the HPA in 1976 (PL 94-360), expanding the inspection program by directing the Secretary of Agriculture to prescribe, by regulation, requirements for the appointment of persons qualified to conduct inspections for the purpose of enforcing the Act. The Designated Qualified Person (DQP) program was established by regulations published in the Federal Register in 1979, and functioned to recruit individuals from the walking horse community to self police the Act. 

The DQP program provides one of the primary mechanisms for detecting sore horses. Horse Industry Organizations (HIOs) with certified DQP programs participate with APHIS in yearly DQP training seminars, refresher clinics, and educational forums. APHIS veterinary medical officers (VMOs) provide instruction and guidance at these sessions, which incorporate classroom training as well as “hands-on” instruction with horses. Regulatory policy, procedures, and methods of inspection are reviewed throughout the year with representatives of the horse industry. Although the law has been in place for over 40 years there is little evidence that this method of self regulation has reduced the practice of soring horses [137,138]. The audience regulated, is not cooperating with authorities and clearly places more value on having a horse display “the big lick” than preventing avoidable pain in horses. The big lick is an exaggerated high stepping gait of competition Tennessee Walking Horses [139].

## 6. Opportunities—Hope for Future

An evolving standard of decency mark the progress of a maturing society [140]. For good reason (fear of tyranny), government is deeply distrusted in Anglo-American tradition. As society becomes more pluralistic, it is more difficult for governments to clearly identify what policy to pursue as there is no clearly defined majority opinion and significant inability to come to consensus what is community decency [93,141,142]. A new law codifies a “new norm” that has developed and established itself in society [2]. A good example of this is the recent passing of many laws enforcing where an individual can and cannot smoke tobacco. The scientific knowledge that smoking killed smokers was available for many decades without the development of any restrictions on individual freedoms. It was the scientific evidence of the harm of second hand smoke to the “innocent” others, lung cancer in non-smokers and the increase in mortality and low birth weight of the newborn that was primary in decreasing the social acceptance of smoking in public. This prior and significant change in social acceptance was necessary to permit laws restricting personal freedom. It is unclear that we have a new societal wide norm for livestock production [143]. 

### 6.1. Soft—Government Regulatory Oversight

Farm policy in North America has been weighted toward unconditional financial support and away from regulation. Recent attempts to implement minimally invasive regulations to enable US cattle traceability have been thwarted by lack of cooperation from the livestock sector [144]. Soft regulation is an approach where future enforcement is threatened after a long period of government incentive programs to enable compliance, such as the European phase in of group housing for sows [145]. Government could regulate the production of livestock in a more precipitous manner as occurred in the sow stall issue in Britain. Hard Regulation with competent enforcement has at least three regressive costs for society. Firstly, the cost of licensing a large farm is the same as a small farm and cost of new animal welfare programs works against survival of small farm operations. Secondly; food is a necessity and if food costs increase incrementally due to new regulations, the future cost of food represents a greater proportion of income burden for poor people than for the wealthy, representing regressive public policy. Finally, resources committed to inspection and enforcement competes with other possible opportunities to improve the welfare of animals and humans. There may be other unforeseen negative consequential outcomes from regulatory approaches.

### 6.2. Tripartite Cooperation

It may seem a simple and obvious request that society wants acceptable levels of protection of farm animals, at an acceptable cost financially and minimal civil rights infringements. In addition to the near total absence of regulatory oversight of livestock production in the USA [146], the methods that concerned citizens can use to better the oversight of animal quality of life on farm are not readily at hand. Perhaps the best approach could be summarized by the 5 principals put forward to resolve some of the intractable issues in Canadian Governance, namely respect, flexibility, balance, cooperation and rule of law [147].

Tripartism is defined as a regulatory policy that fosters greater societal participation represented by Non Government Organizations (NGOs) in the regulatory process in three ways. First, it grants the NGO and all its members’ access to all the information that is available to the regulator. Second, it gives the NGO a seat at the negotiating table with the targeted community (producer organization) and the Policy wing of the enforcement agency when deals are done. Third, the policy grants the NGO the same standing to sue or prosecute under the regulatory statute as the regulator. Tripartism means both opening to NGOs the darkened rooms where the real business of farm regulation and subsidy is transacted and allowing the NGO to operate as a private attorney-general [135]. NGOs do not directly punish the offender or the offending firm; they punish regulators who fail to prosecute firms where prosecution was in the public interest. The NGO functions to oversee and audit the policing function (prevent regulatory capture and shirking) but do not have a financial benefit from prosecution, fines or property confiscation. It would be hard to imagine a society where animal welfare standards were extended to the protection of farm animal production and did not have an associated NGO that pushed politically for the regulation in the first place [146]. 

### 6.3. EFTA Surveillance Authority—A Potential Model

The EFTA (European Free Trade Association) Surveillance Authority monitors compliance with European Economic Area rules in Iceland, Liechtenstein and Norway, enabling those three countries to participate in the European internal market. These rules include the applicable statutory requirements for farm animal welfare assurances that are in place in The European Economic Area (EEA). Reports of the EFTA Surveillance Authority are an example of a review and audit of a functioning animal welfare assurance system. In the 2009 Norway report [148], the main objective of the mission was to assess the application by the Norwegian competent authorities of the EEA legislation regarding methods of production and animal welfare on farms. A particular focus was put on the following areas: (a) the legal and administrative measures in place to implement the legislation requirements, (b) the control framework established and operated by the competent authority to ensure the uniform application of these requirements, (c) other measures to achieve compliance, and (d) the follow-up of controls, including corrective actions.

Using this as a reference document as a template of a national farm animal welfare system the topic of this paper (law and policing) is clearly seen as but a small component of the overall infrastructure that will have to be developed in North America if our society should ever decide to place significant importance on the welfare of livestock in production systems. This paper is primarily directed at sorting out a “Competent Authority” and the problems of field policing would fall into part “b” of the EFTA audit process. 

### 6.4. Farm Animal Welfare as Part of the National-State Veterinary Infrastructure

In anticipation of methods of livestock production becoming a future trade impediment, The OIE has developed international animal welfare standards for animals kept for human use. The OIE is a veterinary organization primarily involved in the veterinary certification of live animals and animal products for international trade [149]. The term “competent authority” is OIE defined as: *the Veterinary Authority or other Governmental Authority of an OIE Member having the responsibility and competence for ensuring or supervising the implementation of animal health and welfare measures, international veterinary certification and other standards and recommendations in the Terrestrial Code in the whole territory* [150]. Their approach presumes that future farm animal welfare/methods of production certification for trade will be delivered by the National-State Veterinary Authority.

The veterinary profession has claimed a leadership role in animal welfare and many individuals work with dedication in shelter practice [151,152] and are a potential resource in the provision of animal welfare standards on farm [153,154,155]. Veterinarians are a highly educated, self regulating profession that does understand the questions of balancing animal welfare with other demands of farm business operation. In addition, those individuals working in the livestock industries are embedded in the communities and meet the general capacity to deliver a form of legitimate “community policing” of animal welfare. Currently private practicing veterinarians deliver many of the federal and state phytosanitary certification services with sufficient integrity to satisfy international agreements on live animal trade, so have some pre-existing legitimacy in enforcement activities. 

The well publicized (in the USA and Canada) 2006 case of killing sows by strangulation, Wiles Hog Farm, Creston OH, the subject of the HBO (Home Box Office) documentary Death on a Factory Farm [156] is not strictly a case of regulatory capture as neither of the two veterinary expert witnesses testifying in court was functioning as a regulatory authority. However; at the trial two veterinarians, declared experts by the court, Dr. Donald Sanders (Ohio State University) and Dr. Paul Armbrecht (career swine practitioner), expressed contradictory opinions on the sow killing methods at the Wiles farm. Dr. Donald Sanders supported the humane euthanasia standards of American Veterinary Medical Association, and the American Association of Swine Veterinarians indicating the strangulation of sows as unacceptable on welfare grounds. Dr. Paul Armbrecht testifying for the defense stated strangulating a sow by lifting her by a sliding chain around the neck attached to a front end loader was humane and a “common industry practice”. This example can be interpreted as practicing veterinarians are at a high risk of regulatory capture as they can become extremely embedded in the industry in which they work. A brief review of hanging as a form of capital punishment will reveal that it was chosen precisely because it was a brutal and a spectacular way for the community to participate in the punishing of the convicted criminal [157,158,159]. No significant moral statement was made by the judiciary in the resolution of this case, perceived as a lost opportunity to many concerned with improving farm animal welfare.

If Canada and the USA accept the OIE opinion that farm animal welfare assurance is a function of the National-State Veterinary Infrastructure, then the sub-national veterinary licensing bodies must act aggressively to evaluate and decrease the possible deficit in the professions’ knowledge and the variance of opinion on animal welfare [160]. In the absence of a competing trans-national identifiable group, the OIE may have significant influence on this question as they currently broker phytosanitary conditions of live animal movement on behalf of the World Trade Organization. 

Departments of agriculture as an enforcement branch have occasionally not retained legitimacy with the citizens. As an example; in 2004 the Swedish Government relieved The Swedish Board of Agriculture (SBA) of the responsibilities to enforce the laws related to animal welfare and human behavior in regards to prohibited treatment of animals [161]. An Animal Welfare Policing Agency independent of the ministry of agriculture was created to enforce and create new animal protection legislation. A growing proportion of the public and the political establishment were of the opinion that the SBA did not give appropriate attention to animal welfare issues; in all likelihood, a perception of regulatory capture. Enforcement of farm animal welfare in Sweden is now at the discretion of the municipalities and county administrative boards, demonstrating a very high level of community participation. 

## 7. Discussion

There is a significant public pressure building in Canada and the U.S. for more modernized legislation and enforcement of the public concerns for farm animal welfare. In theory a legal citation should not be perceived as punishment, but, should function to focus the citizen's attention on a set of habitual behaviors that they may not have fully integrated with the citizen’s own beliefs about relevant social responsibility [162]. With the current absence of a general societal norm applied to farm animal welfare in North America, it is difficult to implement good legislation and a good enforcement program; one that stimulates self reflection by the offender and produces a permanent change in future behavior. 

It is self evident that there is no significant regulatory structure to protect the welfare of farm animals in the USA-Canada meat production complex [119]. In addition we have witnessed some spectacular failures where regulation was attempted. The case of The Horse Protection Act was clearly doomed from the beginning. Where there is no moral alignment between the regulator and the target audience; massive norm-divergence conditions, compliance with non resonate rules cannot be reasonably expected. 

How we as a society initiate discourse on this subject area is a matter of good judgment. For example, two significant papers specifically deal with this fact of absence of law applicable to farm animal welfare in the USA and take highly variant approaches. The first one Engelsman [118], factually correct, approaches the information in what can be described as a naming and shaming dialogue [163]. The second one Mench [17] covers approximately the same material, so also factually correct, but, in a manner much more respectful and considerate of the reality that at least some American citizens work and make a living from livestock husbandry, have pride in their work and have done so for generations. These two papers reflect a problem with a long tradition of regulatory research: What motivates people to comply with the law? A closely related question seeks to address how regulators can best encourage long-term voluntary compliance with their rules and decisions. 

A historic debate in the regulatory literature has been between those who suggest compliance is shaped by implementing harsh sanctions and penalties (the deterrence view), and those who believe that gentle persuasion, identity reflection and cooperation works in securing compliance (the accommodative view) [97]. We have no evidence that either one of these regulatory approaches will ultimately improve the welfare of farm animals; however, based on voluntary compliance models in prisons and in application to general maintaining the peace, there is reason to pursue incremental, respectful and polite dialogue and seek conversion without the sword [92,122].

All policing is local; a member of the competent regulatory authority must be present to inspect, organize the evidence of an offence and present it to the local judiciary having the task of determining justice. Private funding of private policing activities poses challenges to credibility and maintenance of a just and transparent enforcement process, especially in the volatile environment of animal protection. Self funded, highly politicized organizations are not seen as providers of fair policing in farm animal production; they are and will continue to be illegitimate and reflect either two society policing, or predatory policing at best; and aggressive resistance from the agriculture community is assured. 

Globally, as reflected in recent OIE initiatives, animal welfare is being increasingly recognized as a regulatory function of veterinary authorities. National veterinary authorities have limited ability to provide local animal welfare policing. Sub-national veterinary authorities (state and county level) have an opportunity to serve the agricultural community to take a lead in establishing legislation and working out responsible and efficient local reporting, inspection and only when necessary enforcement operations. Doing the job well (quality of decision making) and treating people fairly supports legitimacy of the authority administrating police functions and is critical for success. 

Animal rights groups have reason to perceive that the veterinary community is yet another oppressor of animals and is already captured by the farming community; however agriculturally the veterinary inspector may be the least offensive option to the farming audience. The research on why people obey the law strongly suggests that animal welfare policing services should be provided as a professional public service and not linked with highly politicized self-funding initiatives. Veterinary colleges providing curricula in regulatory veterinary medicine should include the enforcement of animal welfare statutes as an emerging area of the practice of veterinary medicine. 

Norway appears to have gone in a novel direction by creating law that will encourage respect for animals [164]. In the Norwegian system the definition of “unnecessary suffering” in any specific case is not defined by legislation but by a local committee in touch with the common sentiments in the population; what a reasonable Norwegian would expect of his/her fellow citizen. Citizens should see their role not as obedient subjects in a rule-based environment, but as participants in a co-operative effort to operationalize the moral imperatives inherent in animal use for human benefit. The operational policing system is not yet described in the literature.

Strength of both Canada and the USA is that there is currently billions of dollars worth of direct and indirect subsidies going from the citizen taxpayer into agriculture. These cash transfers were originally intended to be in the public interest, however many commentators no longer believe that to be true. It should be possible to divert some of the current unconditional direct payments to farmers to conditional payments to farmers, the conditions being some auditable standard of animal care. Canada cannot morph this current state of animal protection into a national program in the near future as the federal level of government has made strong signals that animal welfare is not included in the National Social Policy. 

Few things are known for certain; however, in Canada the federal government is absolutely not going to initiate any sort of national farm animal welfare initiative in advance of similar actions by their largest trading partner the USA. This has been the historic Canadian position. Compassionate slaughter is legally outside criminal law as there is no evil intent, however most modern countries have a transparent legal or regulatory system to assure the humane slaughter of farm animals. Canada currently has no comprehensive national legal framework or system to assure the protection of animals at the time of slaughter [165,166]. 

In response to societal concern, the United States proclaimed the Humane Methods of Slaughter Act in 1958 [167] and it remains in force as: (Congress) finds that the use of humane methods in the slaughter of livestock prevents needless suffering; results in safer and better working conditions for persons engaged in the slaughtering industry; brings about improvement of products and economies in slaughtering operations; and produces other benefits for producers, processors, and consumers which tend to expedite an orderly flow of livestock and livestock products in interstate and foreign commerce [167,168]. The United States is the primary export market for meat and meat products from Canada. To maintain the appearance of a level playing field for trade, the Canadian Government passed the *Humane Slaughter of Food Animals Act* in 1959; not as a result of any societal concern but to meet the industry needs to have a balanced playing field in relation to US trade. The only abattoirs required to comply were those certified as US export eligible, and the only agency allowed to enforce the Humane Slaughter Act was the meat inspection branch of the Veterinary Inspection Directorate of Agriculture Canada. 

It appears the US is considerably ahead of Canada in potential to implement national farm animal law as they have stronger institutionalism related to the national intergovernmental relations [105]. The US also has a long history of Federal-State-University extension coordination. In addition the USAHA is a forum that meets regularly that can provide discourse on method of livestock production in parallel with the assumed delivery model of the OIE. 

## 8. Conclusion

In a democratic society, the public expects to have its opinion count and as there is a range of opinions on what constitutes the value and quality of animal experiences. With lack of consensus, especially around the welfare of farm animals and the historic special place of agriculture in government policy, significant barriers exist to achieving reasonable oversight of livestock production in Canada and the US. 

Comparing farm animal protection in Canada and the USA with the FAO document [9] standard that the provision of animal protection requires a system with five components; competent authority, advisory function, policing operations, written statutes, and societal engagement; it is nearly zero out of five. Currently in Canada and the USA there is no competent authority. There is no national regulatory body responsible to develop and assure farm animal welfare. In the absence of a responsible representative of society there is no method of providing advice to that authority. Other than animal transportation where there is some regulatory oversight, there is no national operational arm of law enforcement that reaches to the farm level. Animal protection laws in Canada and the USA exclude “accepted farm practices” which are unilaterally decided by the respective industry without the engagement of civil society; so largely there are no laws describing standards to enforce. Finally the engagement of civil society is limited and for mytho-historical reasons current farmers are somewhat excluded from criticism despite the criticism of industrialization of farming as present in the PEW Commission report [169]. 

The fuzzy way forward depends on political will of individual states responding to citizen concerns and start to extend duty of care to farm animals under their animal protection laws. Once a legal framework is in place the competition for limited enforcement resources can begin. Due to scale of production and current ideology of “small” government there will be a critical need to develop a policing system that follows the model of procedural justice and “policing by consent”. The livestock industry is too large, currently too secretive, too geographically dispersed to rapidly or radically deviate from the current laissez-faire economic environment. 
